# Relationship between deltamethrin resistance and gut symbiotic bacteria of *Aedes albopictus* by *16S* rDNA sequencing

**DOI:** 10.1186/s13071-024-06421-3

**Published:** 2024-08-05

**Authors:** Yingbo Sun, Tingting Li, Guofa Zhou, Yunfei Zhou, Yuhong Wu, Jiabao Xu, Jiarong Chen, Saifeng Zhong, Daibin Zhong, Rui Liu, Gang Lu, Yiji Li

**Affiliations:** 1 Key Laboratory of Tropical Translational Medicine of Ministry of Education, School of Basic Medicine and Life Sciences, Hainan Medical University, Haikou, 571199 China; 2 Tropical Diseases Research Center, Department of Pathogen Biology, School of Basic Medicine and Life Sciences, Hainan Medical University, Haikou, 571199 China; 3 Hainan Medical University-The University of Hong Kong Joint Laboratory of Tropical Infectious Diseases, Hainan Medical University, Haikou, 571199 China; 4 Program in Public Health, College of Health Sciences, University of California at Irvine, Irvine, CA 92617 USA; 5 Department of Immunology and Microbiology, School of Basic Medical Sciences, Zhejiang Chinese Medical University, Hangzhou, 310053 Zhejiang China; 6 Department of Infectious and Tropical Diseases, The Second Affiliated Hospital of Hainan Medical University, Haikou, 570311 People’s Republic of China

**Keywords:** *Aedes albopictus*, Deltamethrin, Gut commensal bacteria, *16S* rDNA, Insecticide resistance

## Abstract

**Background:**

*Aedes albopictus* is an important vector for pathogens such as dengue, Zika, and chikungunya viruses. While insecticides is the mainstay for mosquito control, their widespread and excessive use has led to the increased resistance in *Ae. albopictus* globally. Gut symbiotic bacteria are believed to play a potential role in insect physiology, potentially linking to mosquitoes’ metabolic resistance against insecticides.

**Methods:**

We investigated the role of symbiotic bacteria in the development of resistance in *Ae. albopictus* by comparing gut symbiotic bacteria between deltamethrin-sensitive and deltamethrin-resistant populations. Adults were reared from field-collected larvae. Sensitive and resistant mosquitoes were screened using 0.03% and 0.09% deltamethrin, respectively, on the basis of the World Health Organization (WHO) tube bioassay. Sensitive and resistant field-collected larvae were screened using 5 × LC_50_ (lethal concentration at 50% mortality) and 20 × LC_50_ concentration of deltamethrin, respectively. Laboratory strain deltamethrin-sensitive adults and larvae were used as controls. The DNA of gut samples from these mosquitoes were extracted using the magnetic bead method. Bacterial *16S* rDNA was sequenced using BGISEQ method. We isolated and cultured gut microorganisms from adult and larvae mosquitoes using four different media: Luria Bertani (LB), brain heart infusion (BHI), nutrient agar (NA), and salmonella shigella (SS).

**Results:**

Sequencing revealed significantly higher gut microbial diversity in field-resistant larvae compared with field-sensitive and laboratory-sensitive larvae (*P* < 0.01). Conversely, gut microorganism diversity in field-resistant and field-sensitive adults was significantly lower compared with laboratory-sensitive adults (*P* < 0.01). At the species level, 25 and 12 bacterial species were isolated from the gut of field resistant larvae and adults, respectively. The abundance of *Flavobacterium* spp., *Gemmobacter* spp., and *Dysgonomonas* spp. was significantly higher in the gut of field-resistant larvae compared with sensitive larvae (all *P* < 0.05). Furthermore, the abundance of *Flavobacterium* spp., *Pantoea* spp., and *Aeromonas* spp. was significantly higher in the gut of field-resistant adults compared with sensitive adults (all *P* < 0.05). The dominant and differentially occurring microorganisms were also different between resistant larval and adult mosquitoes. These findings suggest that the gut commensal bacteria of *Ae. albopictus* adults and larvae may play distinct roles in their deltamethrin resistance.

**Conclusions:**

This study provides an empirical basis for further exploration of the mechanisms underlying the role of gut microbial in insecticide resistance, potentially opening a new prospect for mosquito control strategies.

**Graphical Abstract:**

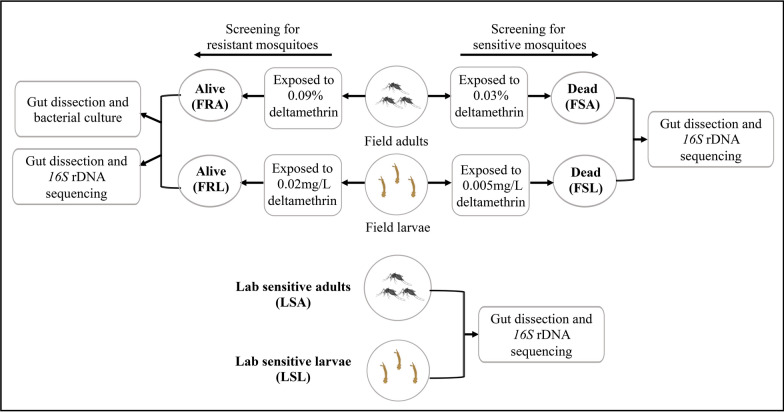

**Supplementary Information:**

The online version contains supplementary material available at 10.1186/s13071-024-06421-3.

## Background

Each year, vector-borne diseases cause hundreds of millions of clinical human cases, resulting in significant morbidity and mortality worldwide. Mosquito-borne diseases, including those transmitted by *Aedes albopictus* (Skuse, 1895), contribute significantly to this burden [1]. *Aedes albopictus*, a highly invasive species, transmits viruses such as the dengue, Zika, and chikungunya [2–4]. Millions of clinical dengue fever cases occur worldwide each year. Dengue fever is most common in Southeast Asia, the western Pacific islands, and Latin America [5]. However, the disease has been spreading to new areas, including local outbreaks in Europe and southern parts of the USA [2, 4, 6]. Outbreaks of Zika have been reported from the Americas, the Caribbean, and parts of Africa and Asia in recent years, and it is a significant challenge to global public health [7, 8]. Local chikungunya fever outbreaks have occurred in several countries in Asia [9]. Unfortunately, there are currently no effective drugs or vaccines for these diseases. Currently, pyrethroid insecticides are commonly used to control the transmission of mosquito-borne viruses due to their low toxicity to mammals and non-target animals. However, widespread and excessive insecticide use has led to resistance in *Ae. albopictus* globally [10, 11].

At present, the known mechanisms of insecticide resistance in mosquitoes can be categorized into four types: epidermal resistance, behavioral avoidance, genetic mutations (e.g., knockdown resistance gene mutation), and enhanced activity of detoxification enzymes [12, 13]. In addition to these known mechanisms, recent studies have highlighted the role of gut bacterial microbiota in insect physiology, metabolism, vector competence, and immune processes [14, 15]. For example, gut microbes in Anopheline mosquitoes metabolize tryptophan, providing protection against parasitic infections [16]. Manipulating gut microbial composition can influence mosquito immune responses and impact the survival of parasites [17]. Furthermore, certain gut bacteria have been found to degrade insecticides and enhance insecticide resistance in insects [18]. Sequencing of gut flora found that the bacteria in the gut of *Megalurothrips usitatus* are involved in various metabolic activities for the degradation of insecticides [19]. In vitro study found that bacteria *Bacillus cereus* and *Pantoea agglomerans*, isolated from the gut of the diamondback moth, degraded indoxacarb insecticide and helped the insect to metabolize the insecticide [20]. Insect gut lactic acid bacteria and probiotics can degrade insecticides and enhance insecticide resistance via hydrolytic enzymes [21]. These studies open a new area for the study of the role of mosquito gut bacteria in insecticide resistance.

However, few studies have investigated the association between gut symbiotic bacteria and insecticide resistance in mosquitoes. In addition, many of these studies used laboratory-selected resistance strain, which may not reflect microbiota community in wild mosquitoes [21–23], because long-term laboratory rearing has led to major physiological changes and nearly fixed food source and environmental condition may select totally different microorganisms inside mosquito body. Moreover, research has indicated that larval and adult mosquitoes may exhibit different resistance mechanisms [24, 25]. Laboratory selections using *Anopheles stephensi* and *Aedes aegypti* have shown that high resistance levels in larvae do not necessarily translate to resistance in adults, suggesting potential differences in metabolic enzyme levels and gut microbiota composition between the two life stages. It has been rarely studied what caused such a difference in insecticide resistance between larval and adult mosquitoes. It is hypothesized that the distinct environmental conditions and microbial communities in larval and adult mosquito guts may trigger physiological changes related to insecticide resistance [26–28]. However, concrete evidence is required to confirm or refute this hypothesis.

This study aimed to comprehensively investigate the relationship between mosquito gut symbiotic bacteria and insecticide resistance. Specifically, we explored whether the gut microbiota of adults and larvae exhibits consistent effects on resistance to insecticides. By examining the shifts in gut microbiota community structure between insecticide-resistant larvae and adults, we sought to identify the dominant bacteria potentially associated with the insecticide resistant and susceptible status. These findings will contribute to the screening of bacteria for future mosquito control strategies targeting insecticide resistance.

## Methods

### Mosquito rearing and insecticide resistance test bioassays

*Aedes albopictus* larvae were collected from 35 aquatic habitats in the field of Haikou City, Hainan Province from June to November 2022. The habitat types were diverse, including coconut shell, plastic basin, metal container, plastic bucket, ceramic jar, foam box, and abandoned tire (Fig. S1). To minimize sampling bias and enhance the generality of the results, larvae from different habitats were pooled and transported to the insectary at Hainan Medical University for rearing. These larvae were reared in their original habitat water without additional food until adult emergence. This approach would maintain the larval gut microbiota similar to natural conditions. Field-insecticide-resistant and field-insecticide-sensitive larvae and adult populations were all screened from these field collected larvae and the gut microbiome was examined using the same larval population. The *Aedes albopictus* laboratory deltamethrin-susceptible strain was donated by the Southern Medical University, Guangdong Province, and maintained at the insectary of Hainan Medical University for 6 years. Laboratory strain larvae were fed with a 1:4 mixture of yeast powder and fish food. Once reached adulthood, mosquitoes were transferred to mosquito cages and provided with a 10% glucose solution as their food source. The insectary was maintained under controlled conditions, at a temperature of 27 ± 2 °C, relative humidity of 60% ± 5%, and a photoperiod of 12:12 h of light:darkness.

**Fig. 1:**
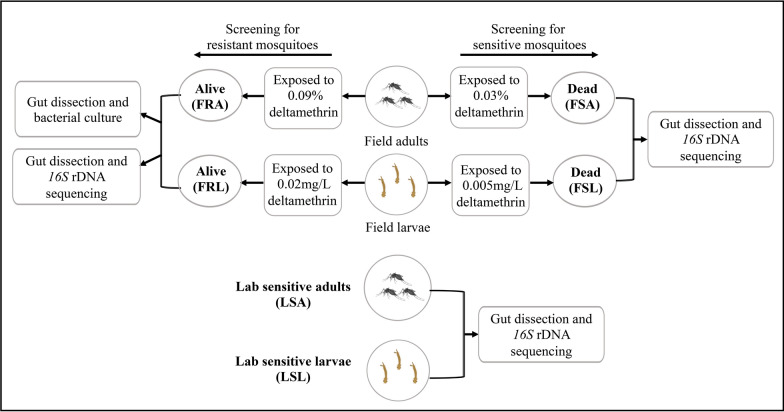
Flowchart for the screening of deltamethrin resistant/sensitive larvae and adult *Aedes albopictus* mosquito gut dissection, and *16S* rDNA sequencing. Field-resistant adults (FRA), field-sensitive adults (FSA), laboratory-sensitive adults (LSA), field-resistant larvae (FRL), field-sensitive larvae (FSL), and laboratory-sensitive larvae (LSL)

The deltamethrin resistance and sensitivity adult *Ae. albopictus* were screened using the World Health Organization (WHO) standard tube bioassay with some modification [29]. Insecticide-impregnated papers and deltamethrin (technical grade 95.95%) were provided by the Chinese Center for Disease Control and Prevention (China CDC). Non-blood-fed, 3–5-day-old F0-generation female mosquitoes were exposed to a 0.09% deltamethrin (3× of standard discriminating concentration) for 1 h. This was conducted with 18 biological replicates, each containing 20 adult mosquitoes. Those that survived the exposure were classified as resistant and used for the gut microbiota study. We utilized a 3× standard diagnostic insecticide dosage for resistant adult mosquito selection, aiming to select individuals with relatively higher resistant levels, anticipating more distinct gut microbiota community structures. Conversely, dead mosquitoes screened with a 0.03% standard discriminating concentration deltamethrin were considered insecticide-sensitive in this study. This was done with 16 biological replicates of 20 adult mosquitoes each. Larval resistance was determined using a modified WHO larvae bioassay method [30]. Third- to fourth-instar field collected larvae were exposed to a 20 × LC_50_ (0.02 mg/L, resulting in 50% mortality) and 5 × LC_50_ (0.005 mg/L) deltamethrin for 24 h. A total of 16 biological replicates were conducted for each dosage with 20 larvae per replicate. Larvae surviving 0.02 mg/L deltamethrin were classified as highly resistant, while those that were killed after 24 h by 0.005 mg/L deltamethrin were considered sensitive for the gut microbiota study. The higher larval dosages aimed to select individuals with higher resistance levels, anticipating more distinct gut microbiota communities. Following deltamethrin treatment, surviving and dead individuals were collected individually for subsequent gut dissection, bacteria culture, and bacterial *16S* rDNA sequencing (Fig. 1). *Aedes albopictus* larval and adult mosquitoes of the laboratory-sensitive strain were not exposed to deltamethrin prior to dissection. Their guts were directly dissected and prepared for bacterial *16S* rDNA sequencing.

### Gut dissection and isolation of *Aedes albopictus* gut bacteria

Prior to larval dissection, insecticide screened larvae were rinsed with sterile water, while adult mosquitoes that underwent screening were frozen at −20 ℃. The larvae and adult mosquitoes were surface-disinfected with 75% alcohol for 1 min before dissected under sterile conditions with the microscope. The dissected guts were rinsed with sterile 1× PBS buffer for 30 s. For subsequent microbiota analysis, 4–7 biological replicates (20 mosquito guts in each replicate) were prepared.

Alive field adult and larvae mosquitoes were utilized for bacteria culture following exposure to a 0.09% deltamethrin film and a 0.02 mg/L deltamethrin solution, respectively. The resistant mosquito gut samples (*n* = 20 mosquito guts per sample) were mixed with 300 µl of sterile water and homogenized using a motor-driven tissue grinder (Sangon Biotech®, China). Subsequently, the bacterial solution was diluted into three different concentrations 10^−1^, 10^−2^, and 10^−3^, and 100 µl of each concentration was streaked onto four types of culture media (brain heart infusion agar, BHI; Luria Bertani, LB; nutrient agar, NA; salmonella shigella medium, SS). All experiments were conducted under sterile conditions.

For each dilution, three replicates were cultured individually under aerobic conditions (at 37 °C) for 24–48 h. After incubation, a single colony was selected from each plate (LB, NA, BHI, SS) and subjected to purification through three consecutive rounds of streaking.

### Bacterial isolation and identification

The DNA from the individual colonies was extracted following the guidelines provided by the rapid bacterial genomic DNA isolation kit (Sangon Biotech®, CatNo.: B518225). The primer sequences used for amplifying the*16S* rRNAwere 27F (5′-AGAGTTTGATCCTGGCTCAG-3′) and 1492R (5′-TACGGCTACCTTGTTACGACTT-3′) [31]. The polymerase chain reaction (PCR) was performed in a 25 µl reaction volume with the following conditions: an initial denaturation step at 95 °C for 5 min, followed by 30 cycles of denaturation at 95 °C for 30 s, annealing at 55 °C for 30 s, extension at 72 °C for 1 min and 30 s, and a final extension step at 72 °C for 10 min. The PCR products were sent to the Beijing Genomics Institution for double-strand sequencing, and the obtained sequences were assembled using SeqMan software (7.1.0 (44.1)). Sequencing data obtained have been deposited in GenBank (accession numbers PP572847–PP572883).

### DNA extraction, *16S* rDNA sequencing sample processing and library construction

Genome DNA of 21 larval samples (420 larval gut) and 14 adult samples (280 adult gut) were extracted using the DNeasy Blood & Tissue Kit (Qiagen, Valencia, CA, USA). The DNA of mosquito gut samples were subjected to sequencing at BGI Shenzhen (Shenzhen, China), targeting the V3-V4 variable region of the *16S* rDNA. The amplification of the targeted region was performed using primers 338F (5ʹ-ACTCCTACGGGAGGCAGCAG-3ʹ) and 806R (5ʹ-GGACTACHVGGGTWTCTAAT-3ʹ), which incorporated multiplex identifier sequences [32, 33]. The PCR reaction conditions were 95 °C for 3 min, 95 °C for 30 s, 55 °C for 30 s, 72 °C for 30 s, and 72 °C for 5 min followed with 30 cycles in a reaction volume of 25 μl. The PCR products were examined using electrophoresis in 1.0% (w/v) agarose gels in TBE buffer (Tris, boric acid, EDTA) stained with Ethidium bromide (EB) and visualized under UV light. Meta amplicon library preparation was conducted using the BGISEQ-500 platform following established protocols [34, 35]: briefly, the PCR product was first denatured into single strands. Subsequently, a cyclization reaction was performed to generate single-stranded circular DNA molecules. This was followed by digestion of uncyclized linear DNA molecules. The single-stranded circular DNA molecules were then amplified through rolling circle replication to form DNA nanoballs (DNBs) containing multiple copies. Finally, the DNBs were immobilized on a high-density DNA nano-chip and sequenced using co-probe anchored polymerization (cPAS) technology.

### Data filtering and sequencing fragment assembly

To ensure an accurate sequence data, the raw data are filtered to obtain high-quality clean data using the following procedure [36]. Initially, truncation of reads was performed for those with an average quality value below 20 over a 30 bp sliding window. Additionally, reads whose lengths were reduced to 75% of their original lengths after truncation were excluded. Subsequently, reads contaminated with adapter sequences, reads containing ambiguous bases (N base), and low-complexity reads were removed. In cases where paired-end reads overlapped, a consensus sequence was generated using Fast Length Adjustment of Short (FLASH) reads, v1.2.11. The criteria for generating the consensus sequence included an overlapping minimum length of 15 bp or a mismatching ratio of the overlapped region not exceeding 0.1. For quality control purposes, the iTools Fqtools fqcheck (v.0.25) tool was employed. Furthermore, connectors and primers were removed using cutadapt (v.2.6), and sequence filtering was performed using readfq (v1.0). Finally, the FLASH (v1.2.11) software was utilized for the final splicing of sequences.

### OTU clustering analyzing

Clustering of spliced high-quality sequences into operational taxonomic unit (OTUs) was accomplished by USEARCH (v7.0.1090) with a 97% threshold through UPARSE, resulting in the acquisition of unique out representative sequences [37]. Subsequently, the removal of chimeras was generated by PCR amplification from OTU representative sequences and filtered by UCHIME (v4.2.40) [38]. Finally, all tags are mapped to the OTU representative sequences utilizing USEARCH GLOBAL to calculate the OTU abundance table. The taxonomic annotation of OUT was performed on the basis of the RDP classifier (v2.2) [39].

### Microbial diversity analysis and differential analysis of KEGG function

Microbial diversity analysis was performed using alpha diversity indices, including the Shannon and Chao1 indices. The Shannon index reflects species diversity and uniformity, while the Chao1 index estimates the number of OTUs present in a sample. Alpha diversity was calculated using Mothur software (v.1.31.2) [40] after normalizing the number of sequences across samples. The alpha diversity of each sample was calculated at 97% similarity. Principal coordinate analysis (PCoA) was conducted on the basis of phylogenetic or count-based distance metrics using QIIME software (v1.80) [41] to visualize similarities or dissimilarities in microbial community composition.

Functional gene composition was assessed using PICRUSt2 software (v2.3.0-b) [42]. Kyoto Encyclopedia of Genes and Genomes (KEGG) information was obtained from the corresponding Greengenes OTU ID. The Wilcoxon or Kruskal–Wallis test was used to identify significant differences in KEGG pathways between insecticide-resistant and insecticide-sensitive groups.

### Statistical analysis

The abundance and species composition were analyzed using the non-parametric Mann–Whitney *U* test to assess the significance of the difference of gut bacteria between the resistant mosquitoes and the sensitive mosquitoes (GraphPad 8.0.2). Alpha diversity indices were used to describe sample diversity, including species Shannon indices and Chao1 indices. Alpha diversity indices were compared using one-way analysis of variance (ANOVA) tests (GraphPad 8.0.2). The Wilcoxon signed-rank test was employed for the statistical analysis of differential genes associated with KEGG functions in insecticide-sensitive and insecticide-resistant mosquitoes. Unweighted and weighted principal coordinate analysis (PCoA) were used to analyze microbial community structure among different populations. The ade4 package in R v3.1.1 was used for PCoA analysis.

## Results

### Sample data correction and filtration

**Fig. 2:**
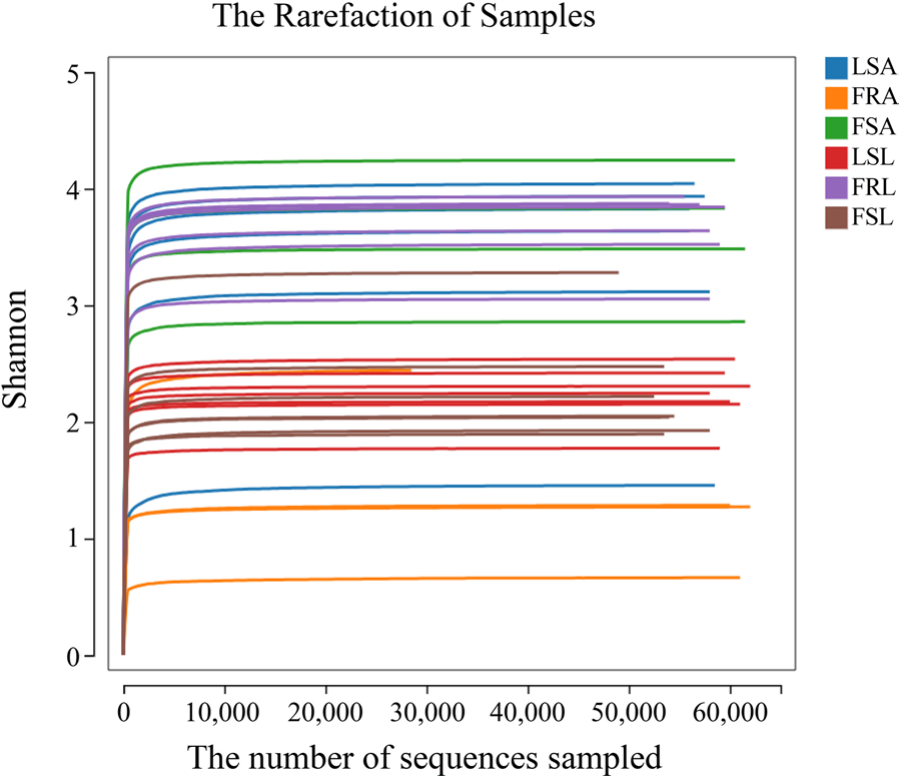
The dilution curves of each mosquito population. Field-resistant adults (FRA), field-sensitive adults (FSA), laboratory-sensitive adults (LSA), field-resistant larvae (FRL), field-sensitive larvae (FSL), and laboratory-sensitive larvae (LSL)

A total of 140 larvae guts (7 replicates) and 120 adults guts (6 replicates) of *Ae. albopictus* laboratory-sensitive strain were analyzed by *16S r*DNA V3-V4 variable region sequencing. A total of 1,012,724 raw sequences were generated, averaging 1446.7 sequences per mosquito gut sample. After the necessary filtering and purification steps, the dataset was refined to a total of 1,006,800 high-quality sequences. Following deltamethrin treatment, 80 field-resistant adult mosquitoes (4 replicates), 140 field-resistant larvae (7 replicates), 80 field-sensitive adult mosquitoes (4 replicates), and 140 field-sensitive larvae (7 replicates) were screened for gut microbiota. A total of 1,719,953 sequences were initially collected and 1,709,107 sequences were obtained after necessary filtering and purification. Subsequently, the analysis of the six sample groups was conducted, and outliers were removed. The resulting dilution curves of the Shannon diversity index were generated for 35 samples (Fig. 2). These curves demonstrated a plateau phase after approximately 5000 sampled sequences, indicating sufficient sequencing depth for subsequent analysis. The sample names, as well as the sequence numbers before and after filtering, are detailed in Table 1.

**Table 1:** The sample raw sequencing data were processed as follows to obtain clean data

Deltamethrin untreated samples				Deltamethrin treated samples									
				Death					Alive				
Strain	Sample name	Raw reads number	Clean reads number	Strain	DC or DFC	Sample name	Raw reads number	Clean reads number	Strain	DC or DFC	Sample name	Raw reads number	Clean reads number
Laboratory-sensitive larvae (LSL)	LSL-1	77,887	77,457	Field-sensitive larvae (FSL)	5 × LC_50_: (0.005 mg/L)	FSL-1	78,070	77,422	Field-resistant larvae (FRL)	20 × LC_50_: (0.02 mg/L)	FRL-1	77,920	77,441
	LSL-2	77,950	77,528			FSL-2	78,247	77,781			FRL-2	78,100	77,603
	LSL-3	78,141	77,698			FSL-3	78,297	77,871			FRL-3	78,149	77,681
	LSL-4	78,469	77,989			FSL-4	78,392	78,003			FRL-4	78,395	77,839
	LSL-5	78,513	78,142			FSL-5	78,732	78,302			FRL-5	78,682	78,128
	LSL-6	77,735	77,271			FSL-6	78,795	78,465			FRL-6	78,719	78,262
	LSL-7	77,824	77,294			FSL-7	77,922	77,459			FRL-7	77,910	77,444
Laboratory-sensitive adults (LSA)	LSA-1	77,502	77,149	Field-sensitive adults (FSA)	0.03%	FSA-1	77,804	77,173	Field-resistant adults (FRA)	0.09%	FRA-1	77,812	77,311
	LSA-2	77,675	77,318			FSA-2	77,975	77,331			FRA-2	77,627	77,289
	LSA-3	78,177	77,561			FSA-3	78,124	77,459			FRA-3	77,807	77,461
	LSA-4	78,077	77,700			FSA-4	78,278	77,605			FRA-4	78,196	77,777
	LSA-5	77,306	76,802										
	LSA-6	77,468	76,891										

Note: DC stands for deltamethrin concentration and DFC stands for deltamethrin film concentration.

### Alpha and beta diversity in the gut microbiome

**Fig. 3:**
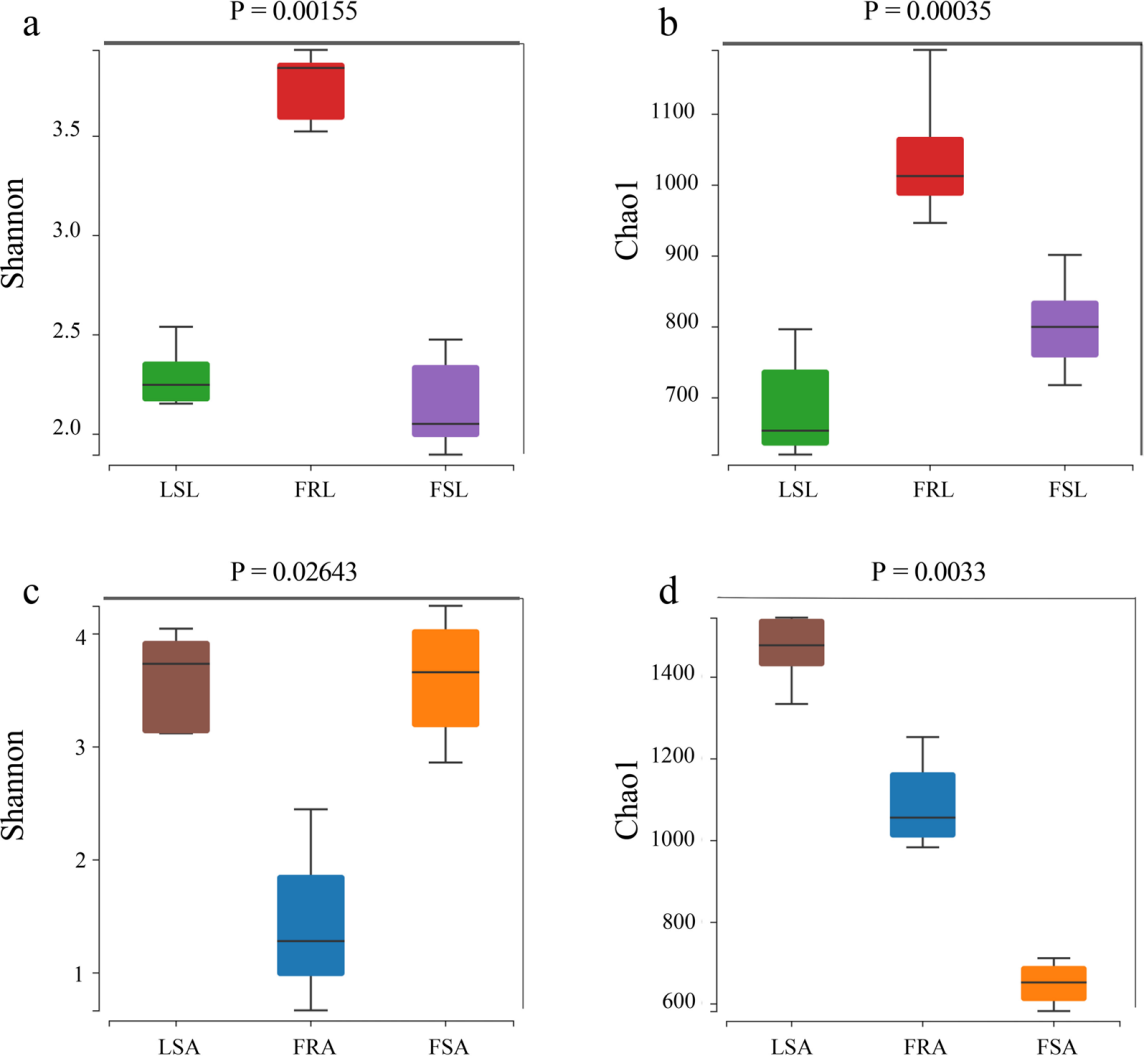
Alpha diversity analysis of gut microbes in deltamethrin-resistant and sensitive larvae and adult *Aedes albopictus* mosquitoes. *Y*-axis represents Shannon or Chao1 index; *X*-axis represent mosquito populations. **a** Laboratory-sensitive larvae (LSL), field-resistant larvae (FRL), field-sensitive larvae (FSL) differences in Shannon indices; **b** Laboratory-sensitive larvae (LSL), field-resistant larvae (FRL), field-sensitive larvae (FSL) differences in Chao1 indices; **c** Laboratory-sensitive adults (LSA), field-resistant adults (FRA), field-sensitive adults (FSA) differences in Shannon indices; **d** Laboratory-sensitive adults (LSA), field-resistant adults (FRA), field-sensitive adults (FSA) differences in Chao1 indices

Sparse curves were drawn from OTU tables to assess the adequacy of sequencing depth in capturing species richness and to facilitate diversity comparisons. Chao1 richness and Shannon diversity were calculated utilizing the gold database (v20110519). Significant differences in species diversity among field-resistant, field-sensitive, and laboratory-sensitive larvae were observed by Shannon (ANOVA, *F*_(2, 18)_ = 36.22, *P* < 0.0001) and Chao1 indices (ANOVA, *F*_(2, 18)_ = 39.80, *P* < 0.0001). Notably, gut microbial diversity and richness were significantly higher in field-resistant larvae compared with susceptible larvae strains (Fig. 3a, b).

Significant differences in species diversity were observed between field-resistant and field-sensitive, laboratory-sensitive adults, as indicated by the Shannon index (ANOVA, *F*_(2, 11)_ = 8.78, *P* = 0.0053) and Chao1 index (ANOVA, *F*_(2, 11)_ = 107.7, *P* < 0.0001). However, the trend observed in adults was opposite to that of larvae, with species richness and diversity in the gut of field-resistant adults being significantly lower than that of sensitive adults with Shannon index (Fig. 3c, d).

**Fig. 4:**
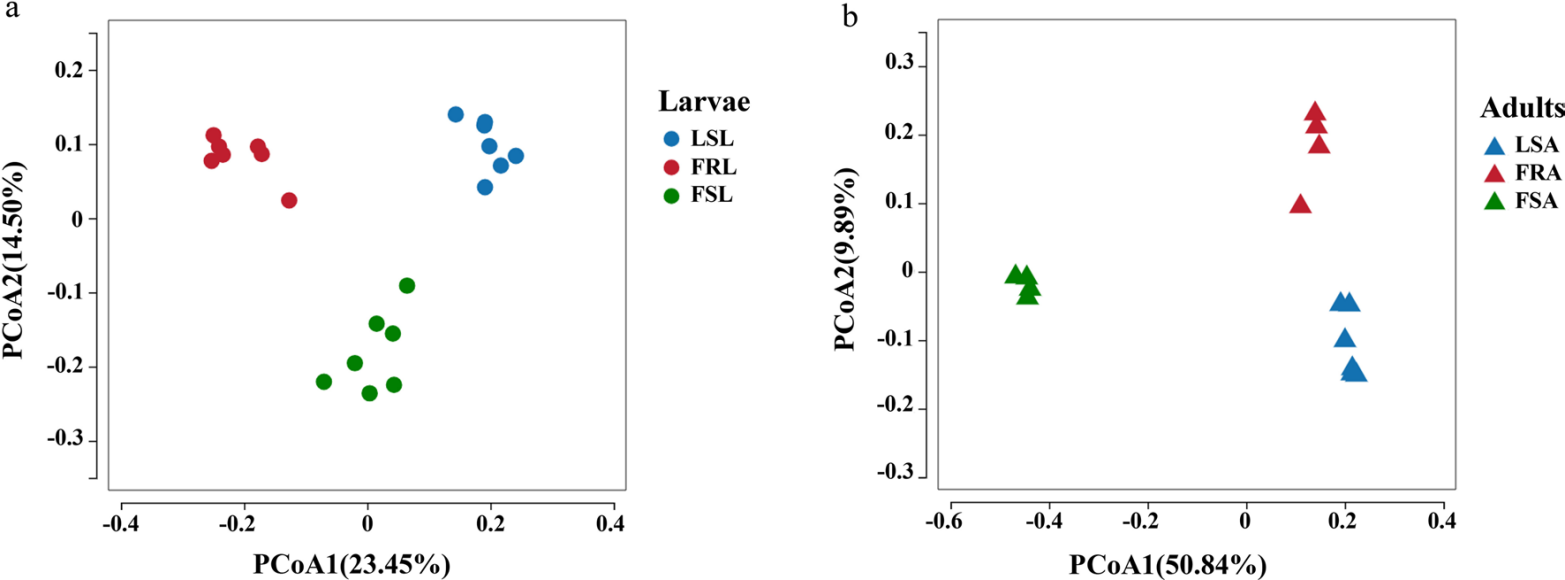
Principal component analysis of gut microorganisms of deltamethrin-resistant and deltamethrin-susceptible larvae and adult *Aedes albopictus* mosquitoes. **a** Represents differences in the PCoA on the basis of the composition of field-sensitive larvae (FSL), field-resistant larvae (FRL), and laboratory-sensitive larvae (LSL). **b** Represents differences in the PCoA between field-sensitive adults (FSA), field-resistant adults (FRA), and laboratory-sensitive adults (LSA)

To assess the discrepancy of gut microorganisms among mosquitoes of field-resistant, field-sensitive, and laboratory-sensitive, beta diversity analysis was conducted using PCoA (Fig. 4), with a full model Bray–Curtis permutational analysis of variance (PERMANOVA) indicating significant differences (*P* < 0.05 for all). PCoA analysis revealed distinct dissimilarities in the gut microbes between field-resistant, field-sensitive, and laboratory-sensitive mosquitoes (Fig. 4a; the larvae Bray *F*_(2, 18)_ = 5.08, *P* < 0.0001, *R*^2^ = 0.36; Fig. 4b; the adult Bray *F*_(2,11)_ = 8.27, *P* < 0.0001, *R*^2^ = 0.60).

### Gut microbiota species abundance

**Fig. 5:**
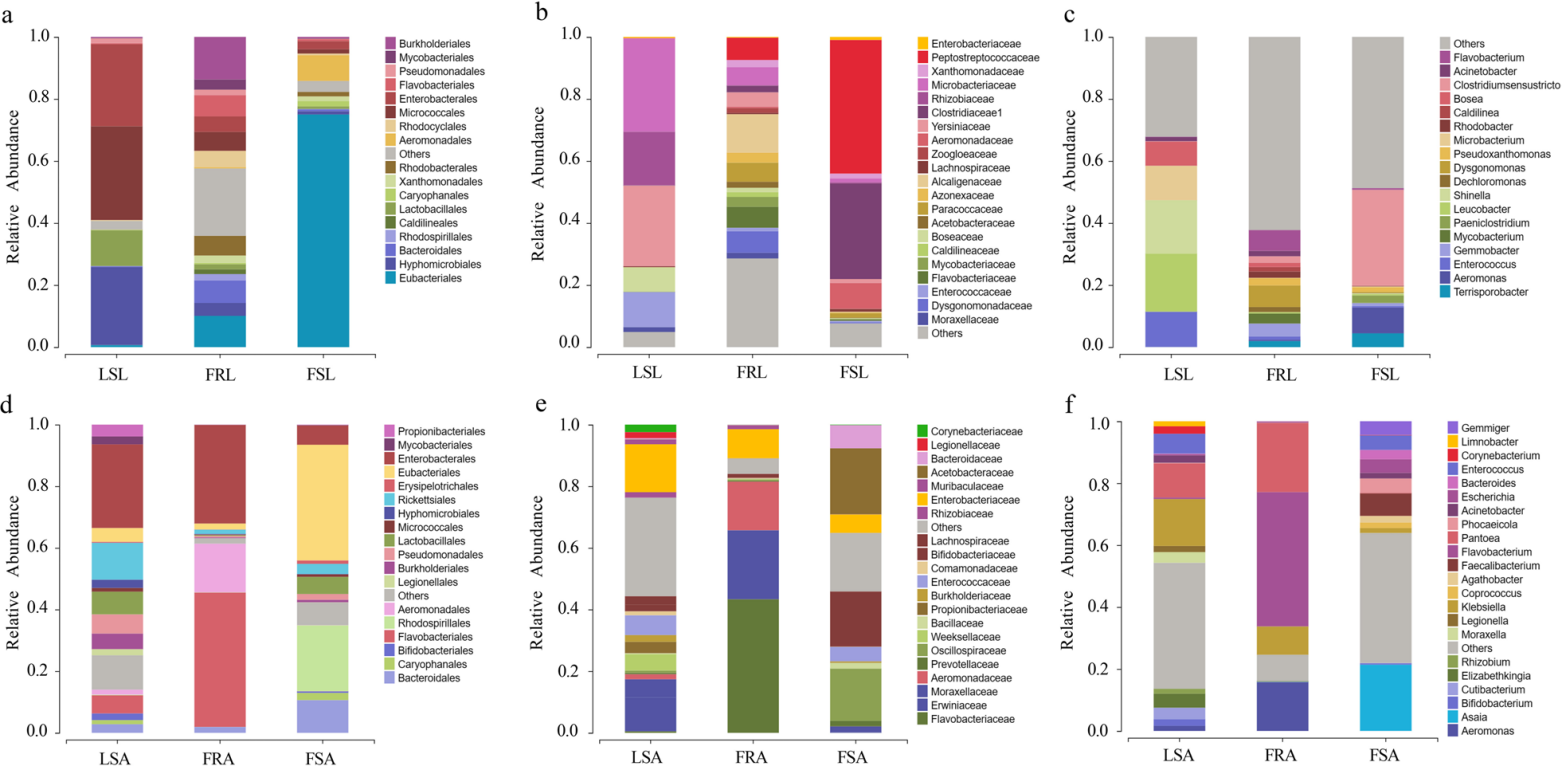
Composition of gut microorganisms of deltamethrin resistant/susceptible larvae and *Aedes albopictus* adult mosquitoes at phylum, genus, and species level. Top panel represents the microbial composition of larvae at the **a**: order; **b**: family; **c**: genus levels. Bottom panel represents the microbial composition of adult mosquitoes at the **d**: order; **e**: family; and **f**: genus levels. Field-resistant adults (FRA), field-sensitive adults (FSA), laboratory-sensitive adults (LSA), field-resistant larvae (FRL), field-sensitive larvae (FSL), and laboratory-sensitive larvae (LSL)

**Fig. 6:**
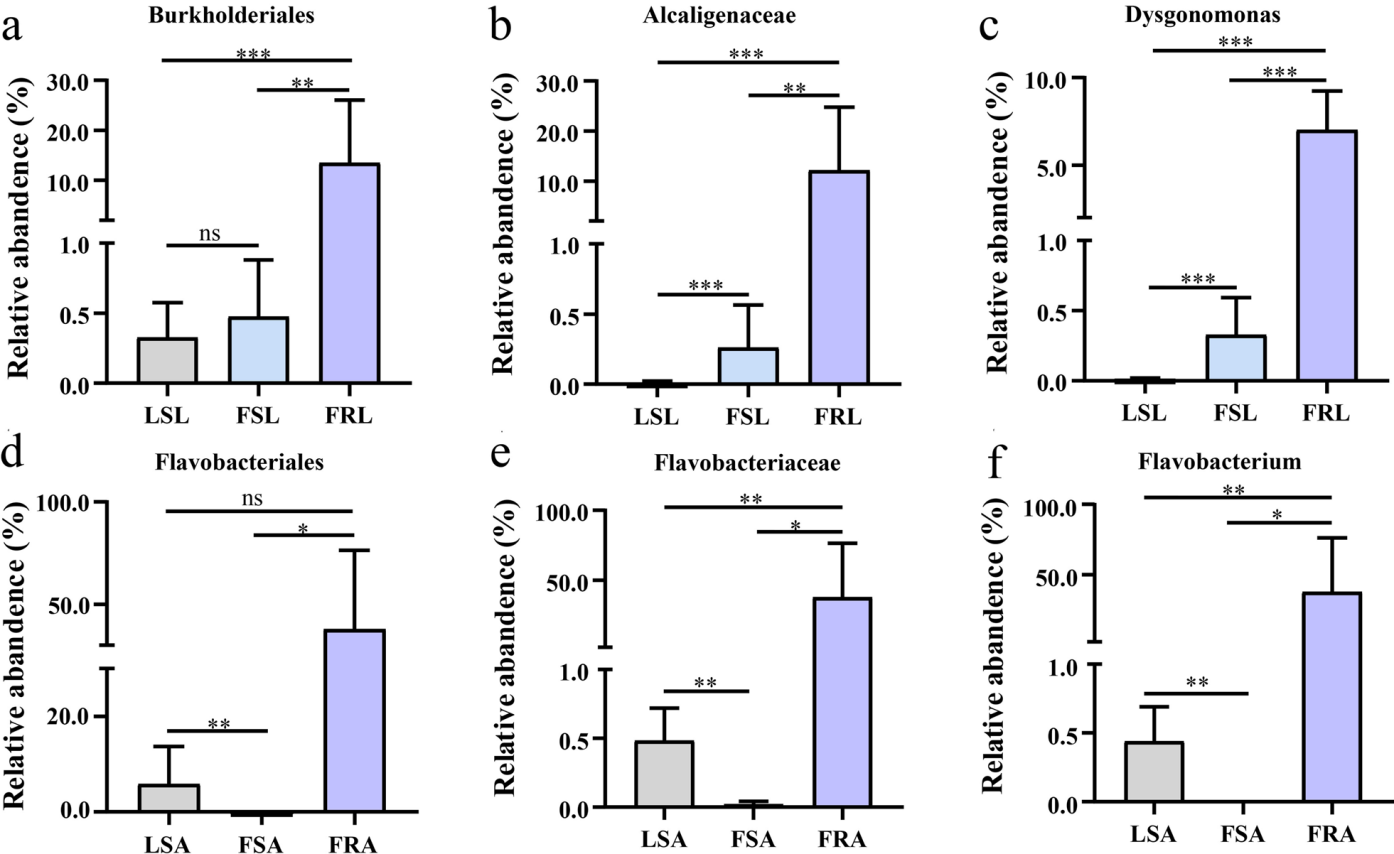
Difference in abundance of gut microorganisms between resistant and sensitive *Aedes albopictus* mosquitoes. Only gut microorganisms with the highest abundance were selected at order, family, and genus levels. Top panel represents the microbial abundance of larvae at the **a**: order, **b**: family, and **c**: genus levels; and bottom panel represents the microbial abundance of adult mosquitoes at the **d**: order, **e**: family, and **f**: genus levels. Field-resistant adults (FRA), field-sensitive adults (FSA), laboratory-sensitive adults (LSA), field-resistant larvae (FRL), field-sensitive larvae (FSL), and laboratory-sensitive larvae (LSL). ^*^P < 0.05, ^**^P < 0.01, and ^***^P < 0.001 all represent significant differences, and ns represents no significant difference

Significant differences were observed in the microbial compositions of field resistant, field sensitive, and laboratory sensitive larvae and adults across various taxonomic levels including order, family, and genus (Fig. 5). In the field-resistant larvae, the bacterial abundances at the order level were primarily annotated to three bacterial phyla: *Burkholderiales, Flavobacteriales, Bacteroidales, Rhodobacterales,* and *Eubacteriales* (Fig. 5a). The relative abundance of *Burkholderiales* in the field-resistant larvae (13.5 ± 4.7%) was significantly higher compared with field-sensitive larvae (Mann–Whitney *U* test, *U*_(14)_ = 1.0, *Z* = −3.0, *P* < 0.01), and laboratory-sensitive larvae (Mann–Whitney *U* test, *U*_(14)_ = 0, *Z* = −3.13, *P* < 0.001). *Eubacteriales* dominated the gut microbiota of field-sensitive larvae, accounting for 74.9 ± 8.1%. *Micrococcales* had the highest abundance in laboratory-sensitive larvae, accounting for 30.2 ± 2.5%. (Fig. 6a). When examining at family level, (Fig. 5b), *Alcaligenaceae* exhibited the highest proportion in the field-resistant larvae (12.2 ± 4.8%), and its abundance was significantly higher than that in the field-sensitive larvae (Mann–Whitney *U* test, *U*_(14)_ = 1.0, *Z* = −3.0, *P* < 0.005) and laboratory-sensitive larvae (Mann–Whitney *U* test, *U*_(14)_ = 0, *Z* = −3.15, *P* < 0.001). *Peptostreptococcaceae* dominated the gut microbiota of field-sensitive larvae, accounting for 42.6 ± 6.1%. *Microbacteriaceae* had the highest abundance in laboratory-sensitive larvae, reaching 30 ± 2.5% (Fig. 6b). At the genus level (Fig. 5c), analysis revealed significantly higher abundances of *Dysgonomonas* spp. (7.0 ± 0.8%) and *Gemmobacter* spp. (4.1 ± 0.7%) in the gut of the field-resistant larvae compared with sensitive larvae (Mann–Whitney *U* test, *U*_(42)_ = 10, *Z* = −4.96, *P* < 0.05). *Clostridiumsensustricto* dominated the gut microbiota of field-sensitive larvae, accounting for 31.3 ± 6.9%. *Leucobacter* had the highest abundance in laboratory-sensitive larvae, reaching 18.7 ± 1.6% (Fig. 6c). Notably, *Flavobacterium* ranked the second most abundant microorganism in the gut of field-resistant larvae (6.7 ± 4.0%), and its abundance was significantly higher compared with field-sensitive and laboratory-sensitive larvae.

Among the field-resistant adults, five bacterial orders, i.e., *Flavobacteriales, Enterobacterales, Eubacteriales, Aeromonadales*, and *Bacteroidales*, exhibited high relative abundance (Fig. 5d). Specifically, the relative abundance of *Flavobacteriales* in the field-resistant adults (37.9 ± 19.2%) was significantly higher than that in the field-sensitive adults (Mann–Whitney *U* test, *U*_(8)_ = 0, *Z* = −2.3, *P* < 0.05). *Eubacteriales* had the highest abundance in the guts of field-sensitive adult mosquitoes, accounting for 37.6 ± 5.2%. *Enterobacterales* had the highest abundance in the guts of laboratory-sensitive adult mosquitoes, accounting for 27 ± 11.5% (Fig. 5d). At the family level (Fig. 5e), *Flavobacteriaceae* had the highest abundance in the field-resistant adults (37.6 ± 19.3%), and its abundance was significantly greater than that in the field-sensitive and laboratory adults (Mann–Whitney *U* test, *U*_(14)_ = 0, *Z* = −2.8, *P* < 0.005). *Acetobacteraceae* had the highest abundance of 21.2 ± 6.6% in the guts of field-sensitive adult mosquitoes. *Enterobacteriaceae* had the highest abundance in the guts of laboratory-sensitive adult mosquitoes, accounting for 15.2 ± 12.4% (Fig. 5e). At the genus level (Fig. 5f), *Flavobacterium* displayed the highest abundance in the field-resistant adults (37.6 ± 19.2%), which was significantly higher than in the field-sensitive strain (Mann–Whitney *U* test, *U*_(8)_ = 0, *Z* = −2.4, *P* < 0.05) (Fig. 6f). Additionally, the abundance of *Pantoea* spp. (20.7 ± 0.15%) and *Aeromonas* spp. (13.6 ± 11.4%) in the gut of the field-resistant adults was significantly higher than that of the sensitive adults (Mann–Whitney *U* test, *U*_(28)_ = 36, *Z* = −2.24, *P* < 0.05). The highest abundance of gut microbes in field-sensitive adult mosquitoes was *Asaia* spp., accounting for 21.2 ± 6.6%. In addition, the highest abundance of gut microbes in laboratory-sensitive adult mosquitoes was *Klebsiella* spp., accounting for 14.8 ± 12.4%.

To compare the dominant gut microorganisms between larvae and adults of field-resistant, field-sensitive, and laboratory-sensitive mosquitoes, we analyzed their abundance at different taxonomic levels. At the order level, *Eubacteriales* emerged as the dominant group in field-sensitive larvae (accounting for 75.0%) and adults (accounting for 37.5%). Notably, the abundance of *Eubacteriales* was higher in larvae than in adults within the field-sensitive mosquito population (Fig. 5a, d). Conversely, at the order level, *Hyphomicrobiales* dominated in laboratory-sensitive larvae (accounting for 25.1%) and adults (accounting for 2.7%) (Fig. 5a, d). At the genus level, *Flavobacterium* prevailed as the dominant group in field-resistant larvae (accounting for 6.7%) and adults (accounting for 43.3%), the abundance of *Flavobacterium* in adults was higher than in larvae (Fig. 5c, f).

### Differences in gut microbiota between resistant and sensitive mosquitoes

**Fig. 7:**
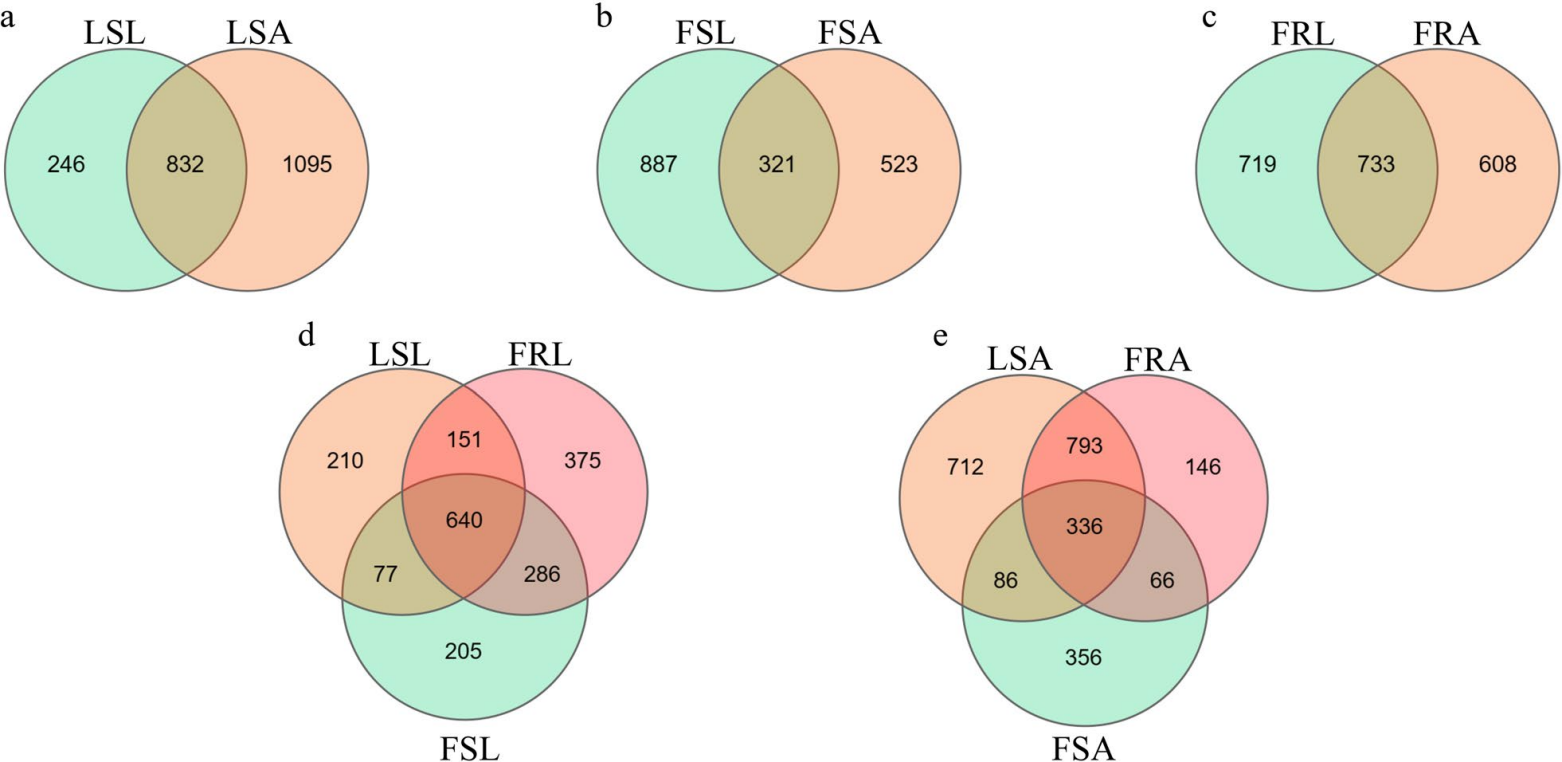
OTU clustering analysis of gut microorganisms of deltamethrin-resistant and susceptible larvae and adult *Aedes albopictus* mosquitoes; **a**: laboratory larvae (LSL) versus laboratory adults (LSA); **b**: field-sensitive larvae (FSL) versus field-sensitive adults (FSA); **c**: field-resistant larvae (FRL) versus field-resistant adults (FRA); **d**: laboratory-sensitive (LSL), field-resistant (FRL), and field-sensitive (FSL) larvae; **e**: laboratory-sensitive (LSA), field-resistant (FRA), and field-sensitive (FSA) adults

To visualize the similarities and differences in microbial operational taxonomic units (OTUs) within the gut of laboratory-sensitive, field-sensitive, and field-resistant strains of mosquitoes, a Venn analysis was conducted. The results showed a total of 2173 OTUs in laboratory-sensitive larvae and adults. Among these, 832 (38.3%) OTUs were shared, 246 (11.3%) OTUs were larval specific, and 1095 (50.4%) OTUs were adult specific (Fig. 7a). Out of a total of 1731 OTUs for field-sensitive larvae and adults, of which 321 (18.5%) OTUs are shared, 887 (51.2%) OTUs are specific to larvae, and 523 (30.2%) OTUs are specific to adults (Fig. 7b). In field-resistant adults and larvae, a total of 2060 OTUs were observed. Among them, 733 (35.6%) OTUs were shared, 719 (35.0%) OTUs were unique to larvae, and 608 (29.5%) OTUs were unique to adults (Fig. 7c).

Similarity and dissimilarity in OTUs were assessed between resistance to sensitive populations in both adults and larvae. The results revealed that the larvae groups encompassed a total of 1944 OTUs (Fig. 7d). Among these, 640 (32.9%) OTUs were shared by all three populations, and 375 (19.3%) OTUs, 205 (10.5%) OTUs, and 210(10.8%) OTUs were specific to the field-resistant, field-sensitive, and lab-sensitive larvae groups, respectively (Fig. 7d). Concurrently, a comparison of 2495 OTUs was performed between the adult groups. The results indicated that 336 (13.5%) of OTUs were shared by all three populations, while 146 (5.8%), 356 (14.3%), and 712 (28.5%) of unique OTUs were unique to the field-resistant and field-sensitive and lab-sensitive adult groups, respectively (Fig. 7e).

### KEGG analysis to predict functional genes

To gain deeper insights into the variations in gene abundance between resistant and sensitive *Ae. albopictus*, we conducted KEGG enrichment prediction analysis. The relative abundance of xenobiotics biodegradation and metabolism-function-related genes [such as Selenocompound metabolism, Pyruvate metabolism, Lipopolysaccharide biosynthesis, Folate biosynthesis, Citrate cycle (TCA cycle), and Butanoate metabolism] were significantly higher in resistance than in sensitive *Ae. albopictus* (Wilcoxon signed-rank test,* Z* = −2.89, *P* < 0.05) (Table S1). In addition, the number of functionally differentiated genes between resistant and sensitive larvae was greater than that of adults (Table S2).

### Cultivable bacteria and strain identification

We successfully isolated and identified a total of 14 genera and 25 species of gut bacteria from the field-resistant larvae samples. Among these, the dominant genera were *Aeromonas* spp., *Bacillus* spp., *Cytobacillus* spp., *Acinetobacter* spp., *Exiguobacterium* spp., *Isoptericola* spp., *Enterococcus* spp., *Leucobacter* spp., *Paenibacill-us* spp., *Staphylococcus* spp., *Priestia* spp., *Serratia* spp., *Staphylococcus* spp., and *Rahnella* spp. (Table 2). In addition, we isolated 5 genera and 12 species of bacteria from the intestines of the field-resistant adult mosquitoes. The main ones were *Bacillus* spp., *Enterococcus* spp., *Kosakonia* spp., *Klebsiella* spp., and *Serratia* spp. (Table 2).

**Table 2:** Isolation and culture to field-resistant *Aedes albopictus* adult and larval gut bacteria in culture media

Organization sources	Classification levels						
	Orders	Families	Species	Media			
				LB	NA	BHI	SS
Field-resistant larvae	*Moraxellales*	*Moraxellaceae*	*Acinetobacter junii*		●		
	*Aeromonadales*	*Aeromonadaceae*	*Aeromonas hydrophila*	●	●		
			*Cytobacillus kochii*	●			
	*Lactobacillales*	*Enterococcaceae*	*Enterococcus gallinarum*		●	●	
	*Bacillales*	*Bacillaceae*	*Lysinibacillus fusiformis*		●		
			*Priestia megaterium*		●	●	
			*Bacillus cereus*	●	●	●	
			*Bacillus infantis*			●	
			*Bacillus megaterium*	●			
			*Bacillus paramycoides*			●	
			*Bacillus sp.*	●	●		
			*Bacillus velezensis*	●		●	
		*Bacillales Incertae Sedis*	*Exiguobacterium aestuarii*		●		
			*Exiguobacterium himgiriensis*	●			
			*Exiguobacterium indicum*			●	
			*Exiguobacterium profundum*	●		●	
		*Paenibacillaceae*	*Paenibacillus polymyxa*		●		
			*Paenibacillus xylanilyticus*	●		●	
		*Staphylococcaceae*	*Staphylococcus epidermidis*		●		
			*Staphylococcus gallinarum*		●		
	*Micrococcales*	*Promicromonosporaceae*	*Isoptericola sp.*	●			
		*Microbacteriaceae*	*Leucobacter chironomi*		●		
	*Enterobacterales*	*Yersiniaceae*	*Rahnella aquatilis*	●	●	●	●
			*Serratia aquatilis*				●
			*Serratia sp.*		●	●	●
Field-resistant adults	*Bacillales*	*Bacillaceae*	*Bacillus amyloliquefaciens*		●		
			*Bacillus cereus*		●	●	
			*Bacillus flexus*			●	
			*Bacillus kochii*		●		
			*Bacillus velezensis*	●		●	
	*Lactobacillales*	*Enterococcaceae*	*Enterococcus sp.*	●			
			*Enterococcus termitis*		●	●	
	*Enterobacterales*	*Enterobacteriaceae*	*Klebsiella pneumoniae*	●			●
			*Klebsiella variicola*	●			
			*Kosakonia radicincitans*	●	●	●	●
			*Kosakonia sp.*			●	
		*Yersiniaceae*	*Serratia marcescens*	●	●	●	

Note: ● means this strain can be well cultured in this medium. BHI, brain heart infusion agar; LB, Luria Bertani; NA, nutrient agar; SS, salmonella shigella medium

## Discussion

The gut microbiota is widely acknowledged as a significant contributor to host development and physiology [43]. In insects, the gut microbiota plays a crucial role in symbiotic digestion of plant-derived polysaccharides, the degradation of toxic compounds such as insecticides, and the establishment of colonization resistance against pathogens [44–47]. While the precise molecular mechanisms underlying host–microorganism interactions remain largely unknown, mounting evidence suggests a close association between the gut microbiota and host resistance to insecticides [43, 48]. In our study, we observed a significantly higher diversity of gut microbes in field-resistant larvae of *Ae. albopictus* compared with field-sensitive and laboratory-sensitive larvae, consistent with previous research [22]. These findings suggest a potential relationship between the diversity of gut microbes in *Ae. albopictus* larvae and their resistance to deltamethrin. Additionally, we found a higher abundance of *Dysgonomonas*, a genus of bacteria, in the gut of field-resistant larvae compared to deltamethrin-susceptible larvae. *Dysgonomonas*, which is Gram-negative, non-motile, and parthenogenetic anaerobic cocci, was initially isolated from human stools and wounds [49, 50]. Recent *16S* rRNA sequencing studies have revealed its wide distribution in terrestrial environments, with enrichment observed in various insect systems, including honeybees and Aedes [51, 52]. Further investigations are required to determine whether the higher abundance of *Dysgonomonas* in resistant Aedes larvae is the result of insecticide selection pressure.

Previous studies have indicated that the accumulation of microorganisms in larval guts primarily originates from plankton in water, soil, and the surrounding environment [53]. A comparison of gut microbes between field-sensitive and laboratory-sensitive Aedes mosquito larvae revealed that *Clostridiumsensustricto* and *Aeromonas* spp. were more abundant in the gut of field-sensitive larvae, and both were found in soil and water in the environment [54, 55]. However, few microbes from the external environment were detected in the gut of laboratory-sensitive larvae. Field-caught mosquitoes exhibit greater gut microbial diversity compared with their laboratory-reared counterparts [56]. This difference is likely attributed to the broader range of microbial communities encountered by mosquitoes in field environments, providing them with more opportunities to acquire diverse microorganisms. Consequently, studying the gut microbiota of field-caught mosquitoes offers a more representative and realistic perspective on their microbial composition compared with laboratory-reared colonies.

Interestingly, our study revealed that highly resistant *Ae. albopictus* adults had lower gut microbial diversity compared with sensitive adults, which represents a novel finding. Two possible explanations can account for this difference. Firstly, the two populations may have been reared under different environmental conditions, which was not the case in the current study. Alternatively, due to the resistance selection, microorganisms that support resistance development may have cumulated, whereas those negatively associated with resistance were eliminated or suppressed, resulting in reduced diversity and an enrichment of microorganisms that promote resistance development. Notably, we found high abundance of *Flavobacterium* in the gut of both the field-resistant adult and larvae mosquitoes, which may be associated with insecticide resistance in mosquitoes. Previous studies have also demonstrated that exposure to *Flavobacterium* and *Paenibacillus* in larvae and adults is associated with lipid metabolism remodeling, increased lipid metabolism, increased lipid storage, and enhanced starvation resistance [28]. Whether this is linked to insecticide metabolic resistance in mosquitoes needs to be further investigated.

Furthermore, our study also revealed a lower microbial diversity in the gut of resistant adult mosquitoes compared with resistant larval microbiota. Similar findings have been reported in a study of field-collected *An. albimanus* [57]. This difference could potentially be attributed to variations in their living environment and differences in resistance mechanisms between larvae and adults. Selection studies in *An. stephensi* and *Ae. aegypti* have indicated that even highly resistant larvae populations may exhibit limited resistance in emerged adults when exposed to the same insecticides [26, 27], indicating possible difference in their resistance mechanisms between larval and adult mosquitoes. With larvae relying on their habitat for food while adult mosquitoes require sugar for survival and blood meals for female oviposition, these may make resistant mosquitoes accumulate different microorganisms, thus potentially leading to different resistance mechanisms. We must note that the cumulation of resistance related and reduced abundance of insecticide sensitive microorganisms in adult mosquitoes likely lead to the lower microorganism diversity in resistant adult mosquitoes, but further in vitro or even in vivo confirmations are needed. In summary, gut microorganism diversity and dominant organisms may be key indicators determining the resistance mechanisms in mosquitoes.

The *Asaia* spp. was detected in the intestines of sensitive adults in the field-caught *Ae. albopictus* in the current study. Previous study found *Asaia* strains in the intestines of *Anopheles* spp. and its presence of pyrethroid hydrolase genes, suggesting that *Asaia* may be involved in insecticide resistance in mosquitoes [58, 59]. The introduction of *Asaia* activates basal immunity in mosquitoes and reduces malaria parasite development in *An. stephensi*, confirms *Asaia*’s potential usage for mosquito control [60]. The role of *Asaia* in insecticide resistance in mosquitoes is worth further investigations.

Strain identification could be made more directly and objectively by bacterial isolation and culture. Isolated from resistant larvae gut, *Acinetobacter junii* is a recognized petroleum-explicating bacterium found in petroleum-contaminated environments. *Bacillus cereus* had also been isolated from resistant larvae gut, and a strain of heavy metal-resistant bacterium Bacillus cereus BCS1 has been found to degrade pyrethroids in a soil–plant system [61, 62]. Both bacteria have the potential to degrade organic matter and may help mosquitoes metabolize deltamethrin and other organic insecticides. However, most of the bacteria in the mosquito gut cannot be isolated or is very difficult to isolate and culture under laboratory conditions.

The differential gene analysis revealed that the metabolic function of the gut microorganisms in resistant mosquitoes was stronger than in sensitive mosquitoes, which may be an adaptive change to the external environment to maintain mosquito homeostasis [63]. At the same time, we found that the metabolic function genes of larval gut flora were stronger than that of adults, according to KEGG results, probably due to the reason that the larval gut flora is more complex and diverse than that of adults and may have more opportunities to participate in the insecticides metabolism.

In our study, larvae were collected from diverse aquatic habitats and pooled for subsequent resistance screening, sensitive mosquitoes selection, and bacteria isolation. This approach aimed to reduce sampling bias and increase the generality of the study results. Different habitats harbor distinct microbial communities, which can contribute to the larval food sources. Mixing larvae ensured a more representative sample of the overall microbial diversity present in the field. For mosquito gut bacteria culture, a variety of media were used, including LB, BHI, and NB. While most gut bacteria can be isolated and cultured in LB medium, some require richer nutrients found in BHI and NB. This approach aimed to maximize the isolation of diverse bacterial species.

This study presents limitations, but it also contributes to the understanding the role of commensal bacteria in mosquito resistance regulation. One of the limitations is the selection method for resistant mosquito populations. Various approaches exist, including standard WHO or CDC bioassays [64], as well as modified standards. For instance, Omoke et al. employed a 5× standard insecticide dosage [65], while Pelloquin et al. used a combination of 3× standard diagnostic insecticide dosage and extended holding time (72 h instead of 24 h) [66]. In this study, we utilized a 3× standard diagnostic insecticide dosage for adult mosquito selection and a 20× standard concentration for larval population selection. The aim was to isolate individuals with relatively higher resistant levels, anticipating more distinct gut microbiota community structures. We hypothesized that selecting resistant mosquitoes with higher insecticide concentrations would reduce microbial diversity on the basis of the selection–cumulation principle [67]. This approach aimed to identify the highly differentially occurring microorganisms associated with resistance development, if they exist. Determining the optimal method for selecting insecticide-resistant mosquitoes remains an area for future research. Using a 3× diagnostic dosage (0.09% deltamethrin) in this study yielded promising results, demonstrating contrast difference in gut microbiota community structure between resistant and susceptible adult mosquitoes. However, caution is required when using higher diagnostic dosage for resistant mosquito selection. Generally, using higher dosages for resistant mosquito population selection is preferable to standard diagnostic dosage, as low resistance mosquitoes may not exhibit significantly different microbial communities compared with susceptible ones. Furthermore, in areas with low insecticide resistance levels, a 3× diagnostic dosage may not select any highly resistant individuals. Therefore, it is recommended to conduct a standard dosage resistance test first to determine the appropriate dosage for insecticide resistant mosquito selection.

Another limitation is that we did not test the functions of the differentially occurring microorganisms or their potential roles in enhancing metabolic enzyme activities. Future work will focus on functional characterization of these microorganisms and their potential roles in mosquito larval and adult insecticide resistance. Nonetheless, examining the potential differences in the roles of gut microbiota of resistant and susceptible *Ae. albopictus* larvae and adults collected from the field would provide valuable insights into the mechanisms by which gut microbiota influence resistance in adult mosquitoes and larvae. In this study we employed different numbers of replicates for population selections of different insecticide resistance. In most if not all insecticide resistance studies, three replicates are standard and minimum. Using more replicates will increase the stability of mortality data and the power to detect differences between resistant and susceptible populations. We tried to increase the number of replicates as much as possible to enhance statistical power. While increasing replicates for laboratory strains is relatively straightforward, field-collected mosquitoes present logistical challenges. It requires considerable effort to collect and rear sufficient larvae to generate a large number of adults, and there is a balance between number of replicates and age consistency. In addition, unforeseeable issues can arise during the process. Therefore, we ended up with different numbers of replicates for different experiments. Ideally, using the same number of replicates throughout the study would be preferable. However, with a minimum of four replicates used, the test results were not compromised.

## Conclusions

The community composition and structure of mosquito gut microbiota play an important role in the development of insecticide resistance. In *Aedes albopictus*, the diversity of gut microorganisms in deltamethrin-resistant larvae and adults exhibited contrasting patterns, potentially due to their adaptation to different environmental conditions. The reduced microbiota diversity observed in insecticide-resistant mosquitoes compared with their susceptible counterparts is likely attributed to the selection–cumulation effect. This effect suggests that microorganisms associated with resistance are selectively enriched and accumulated. Specific microorganisms, such as *Flavobacterium*, *Acinetobacter junii*, and *Bacillus cereus*, have been identified as potential deltamethrin microbials metabolizers in *Ae. albopictus*. Further research is needed to determine whether the overall diversity and structure of gut microorganisms or the abundance of specific microorganisms drives the development of insecticide resistance in mosquitoes.

### Supplementary Information

Supplementary Material 1. Fig. S1. Various aquatic habitats for filed collection of *Aedes albopictus* larvae; a: coconut shell; b: plastic basin; c, d: metal container; e: plastic bucket; f: ceramic jar; g: foam box; h: abandoned tire.

Supplementary Material 2. Table S1. Differences in KEGG functional pathways between deltamethrin-resistant and sensitive *Aedes albopictus* in the field.

Supplementary Material 3. Table S2. Differences in KEGG functional pathways in adult and larval *Aedes albopictus* resistant and sensitive to deltamethrin in the field.

## Data Availability

Sequence data that support the findings of this study have been deposited in the Sequence Read Archive (NCBI) with the primary accession code PRJNA1094869. [http://www.ncbi.nlm.nih.gov/bioproject/1094869].
