# Supplementary figures and images for: Relationship between deltamethrin resistance and gut symbiotic bacteria of Aedes albopictus by 16S rDNA sequencing
Source: Parasit Vectors. 2024 Aug 5;17:330. doi: 10.1186/s13071-024-06421-3 (PMC11299273; doi:10.1186/s13071-024-06421-3)

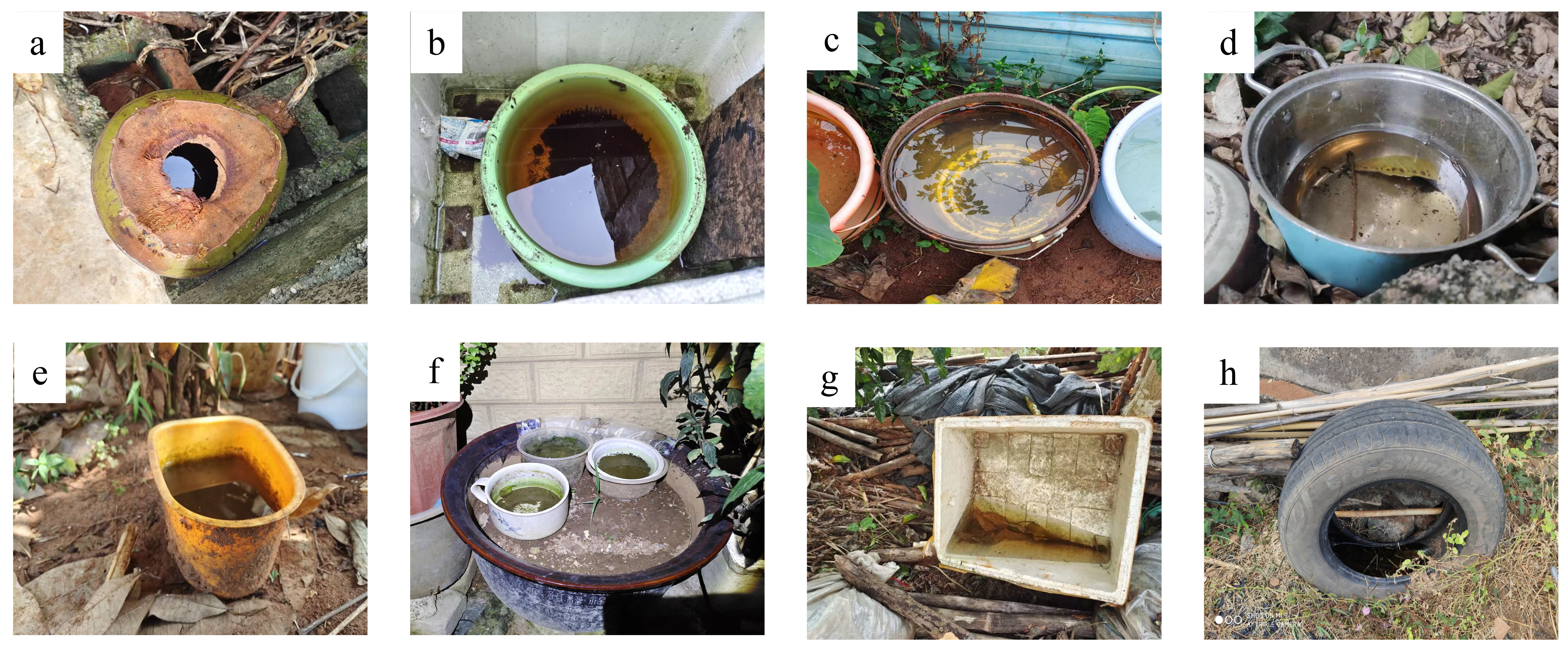

Supplement: Supplementary file 1 — Supplementary Material 1. Fig. S1. Various aquatic habitats for filed collection of Aedes albopictus larvae; a: coconut shell; b: plastic basin; c, d: metal container; e: plastic bucket; f: ceramic jar; g: foam box; h: abandoned tire. [file 13071_2024_6421_MOESM1_ESM.jpg]
