# Supplementary material for: Relationship between deltamethrin resistance and gut symbiotic bacteria of Aedes albopictus by 16S rDNA sequencing
Source: Parasit Vectors. 2024 Aug 5;17:330. doi: 10.1186/s13071-024-06421-3 (PMC11299273; doi:10.1186/s13071-024-06421-3)
Supplement: Supplementary file 2 — Supplementary Material 2. Table S1. Differences in KEGG functional pathways between deltamethrin-resistant and sensitive Aedes albopictus in the field. [file 13071_2024_6421_MOESM2_ESM.docx]

Table S1. Differences in KEGG functional pathways between deltamethrin-resistant and sensitive *Aedes albopictus* in the field

| KEGG functional pathway | FRA (%) | FSA (%) | P value | KEGG functional pathway | FRL (%) | FSL (%) | P value |
| --- | --- | --- | --- | --- | --- | --- | --- |
| Selenocompound metabolism | 1.337 | 1.056 | 0.03 | Valine, leucine and isoleucine biosynthesis | 2.185 | 1.583 | 0.003 |
| Pyruvate metabolism | 1.317 | 1.149 | 0.03 | C5-Branched dibasic acid metabolism | 1.99 | 1.125 | 0.002 |
| Lipopolysaccharide biosynthesis | 1.262 | 0.681 | 0.03 | Synthesis and degradation of ketone bodies | 1.904 | 0.92 | 0.002 |
| Folate biosynthesis | 1.19 | 0.921 | 0.03 | Biosynthesis of vancomycin group antibiotics | 1.633 | 0.917 | 0.002 |
| Citrate cycle (TCA cycle) | 1.114 | 0.862 | 0.03 | Pantothenate and CoA biosynthesis | 1.573 | 1.329 | 0.021 |
| Butanoate metabolism | 0.916 | 0.764 | 0.03 | Streptomycin biosynthesis | 1.336 | 1.004 | 0.015 |

FRL: Field resistant larva; FSL: Field sensitive larva; FRA: Field resistant adult; FSA: Field sensitive adult; %: Significant difference function relative abundance; P value < 0.05 is significantly different.
