# Supplementary material for: Relationship between deltamethrin resistance and gut symbiotic bacteria of Aedes albopictus by 16S rDNA sequencing
Source: Parasit Vectors. 2024 Aug 5;17:330. doi: 10.1186/s13071-024-06421-3 (PMC11299273; doi:10.1186/s13071-024-06421-3)
Supplement: Supplementary file 3 — Supplementary Material 3. Table S2. Differences in KEGG functional pathways in adult and larval Aedes albopictus resistant and sensitive to deltamethrin in the field. [file 13071_2024_6421_MOESM3_ESM.docx]

Table S2. Differences in KEGG functional pathways in adult and larval *Aedes albopictus* resistant and sensitive to deltamethrin in the field

| KEGG function pathway | FSL (%) | FSA (%) | P value | KEGG function pathway | FRL (%) | FRA (%) | P value |
| --- | --- | --- | --- | --- | --- | --- | --- |
| Bacterial chemotaxis | 2.715 | 1.425 | 0.011 | Valine, leucine and isoleucine biosynthesis | 2.185 | 1.804 | 0.011 |
| Flagellar assembly | 1.906 | 0.892 | 0.011 | Pantothenate and CoA biosynthesis | 1.573 | 1.453 | 0.011 |
| D-Alanine metabolism | 1.809 | 1.422 | 0.011 | Geraniol degradation | 1.242 | 0.671 | 0.018 |
| Secondary bile acid biosynthesis | 1.476 | 0.86 | 0.018 | Fatty acid degradation | 1.172 | 0.68 | 0.011 |
| Lipoic acid metabolism | 1.451 | 0.919 | 0.047 | Butanoate metabolism | 1.17 | 0.916 | 0.018 |
| Drug metabolism - other enzymes | 1.375 | 1.067 | 0.011 | beta-Alanine metabolism | 1.027 | 0.72 | 0.011 |

FSL: Field sensitive larva; FSA: Field sensitive adult; FRL: Field resistant larva; FRA: Field resistant adult; %: Significant difference function relative abundance; P value < 0.05 is significantly different.
